# Label-Free Detection of Single Living Bacteria via Electrochemical Collision Event

**DOI:** 10.1038/srep30022

**Published:** 2016-07-20

**Authors:** Ji Young Lee, Byung-Kwon Kim, Mijeong Kang, Jun Hui Park

**Affiliations:** 1 Korea Advanced Institute of Science and Technology, Department of Chemistry, Daejeon, 34141, South Korea; 2 Sookmyung Women’s University, Department of Chemistry, Seoul, 04310, South Korea; 3 University of Maryland, Fischell Department of Bioengineering, College Park, MD 20742, USA; 4 Chonbuk National University, Department of Chemistry Education and Institute of Fusion Science, Jeonju 54896, South Korea

## Abstract

We detected single living bacterial cells on ultramicroelectrode (UME) using a single-particle collision method and optical microscopic methods. The number of collision events involving the bacterial cells indicated in current-time (i-t) curves corresponds to the number of bacterial cells (i.e., *Escherichia coli*) on the UME surface, as observed visually. Simulations were performed to determine the theoretical current response (75 pA) and frequency (0.47 pM^−1^ s^−1^) of single *Escherichia coli* collisions. The experimental current response (83 pA) and frequency (0.26 pM^−1^ s^−1^) were on the same order of magnitude as the theoretical values. This single-particle collision approach facilitates detecting living bacteria and determining their concentration in solution and could be widely applied to studying other bacteria and biomolecules.

Single-particle collisions have attracted considerable attention for over a decade because this technique provides important information on single-particle properties, such as size, concentration, surface charge, and lifetime^1^. Initially, micro-/nanoscale beads composed of non-conducting rigid materials were employed to demonstrate this method’s working principle, which is based on blocking the flux of redox species toward an ultramicroelectrode (UME) through the collision of single particles at the UME, thereby decreasing the electrode reaction^2^,^3^. Interesting properties of various rigid particles have been observed using this unique analytical platform^4^,^5^,^6^.

Recent publications have broadened the range of materials that can be studied via this single-particle-collision-based technique, including soft particles (e.g., emulsions)^7^,^8^. Since the introduction of the emulsion particle collision technique, biomolecule-detection studies investigating viruses, proteins, and DNA have been reported^9^,^10^. Indeed, this technique enabled us to characterize individual particles consisting of such soft materials^11^. Interesting features of various single biomolecules have been observed^12^,^13^, but label-free detection of single living microbes has not been reported to date. Because a single unit of living matter (e.g., a bacterium) can proliferate over time and ultimately generate significant phenomena (e.g., disease), the rapid, real-time detection of such units via single-particle-collision-based analysis can provide valuable information about and/or control over living microbes.

The detection and quantification of bacteria are in high demand in the fields of clinical pathology, food science, and biology. However, it is difficult to monitor or capture living bacteria, which are small and move by swimming, swarming, gliding, and twitching in solution^14^. Furthermore, the rapid self-proliferation of living cells increases the difficulty of estimating their exact concentrations^15^. Currently, a variety of approaches are available to measure bacteria in samples^16^,^17^: optical density, microscopy techniques, and cell cultivation^18^,^19^,^20^,^21^. Although each technique has unique advantages, they are associated with some limitations that hinder their wide application. For example, using cultivation methods, the bacterial concentration can be obtained in ~5 days; thus, these methods are time consuming. Spectrophotometrically measuring the optical density and correlating it with the bacterial concentration is rapid and simple, but the specific response factors for the investigated bacteria and media must first be calculated; additionally, the sensitivity and accuracy of this method are not sufficiently high. Microscopic counting is a straightforward method to quantify bacteria. However, this method cannot be used for small (less than 2 μm) bacteria. Therefore, a fast and reliable technique able to detect specific bacteria and determine their concentrations is urgently needed.

Here, we report the detection of a single living bacteria on a UME. The concentration of living bacteria can be measured based on the number of captured cells at the UME surface, and the electrochemical signal can be used to estimate the size of the bacteria. *Escherichia coli (E. coli*) which is a rod-shaped, gram-negative bacteria with a length of ca. 2 μm was chosen as a model bacteria in this work. The mobile living bacteria were detected at the UME according to two sequential strategies: 1) electrophoretic migration and 2) blockage of the electroactive area. To observe an *E. coli* collision event, a redox species is continuously oxidized at the UME surface (Fig. 1). When the ferrocyanide (Fe(CN)_6_^4─^) is oxidized at the electrode surface, a positive electric field is developed by the steady-state current flow. Therefore, the negatively charged *E. coli* is attracted to the UME surface through electrophoretic migration. When an *E. coli* collides with and then attaches to the UME surface, the level of the steady-state current decreases immediately because the flux of the redox species is blocked by the *E. coli*. Therefore, a staircase current response is observed (Fig. 1).

## Results and Discussion

### Determination of experimental conditions for electrochemical collision events

**Figure 2:**
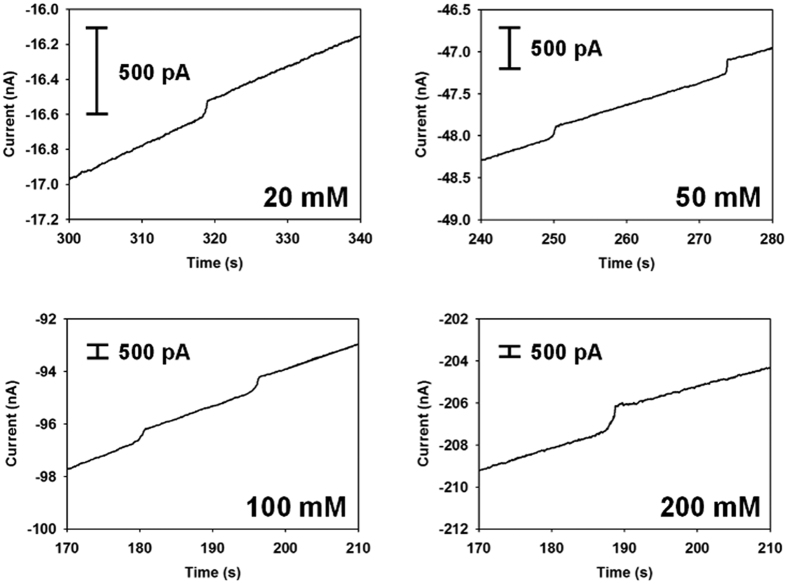
i-t curves of single *E. coli* collisions at a carbon fiber-UME (C-UME). The solution contains 53 fM *E. coli* and various concentrations of Fe(CN)_6_^4−^. The potential was +0.6 V applied at 0 s.

To distinguish a single living microbial collision event based on UME surface blockage, the experimental conditions must be carefully chosen. First, the solution composition should be adjusted. The single-particle blocking experiment was typically performed in the presence of a high concentration of redox species (e.g., 400 mM Fe(CN)_6_^4─^) to obtain a high current intensity^7^. However, such high concentrations of redox species may cause unexpected damage to the living *E. coli*. Therefore, relatively low Fe(CN)_6_^4─^ concentrations able to produce observable collision responses were investigated. Four different concentrations of redox species were tested for single *E. coli* collisions. As shown in Fig. 2, staircase current responses were observed upon single *E. coli* collision for all studied concentrations, and the current step height was proportional to the redox species concentration (Supplementary Fig. S1). In the presence of 20 mM Fe(CN)_6_^4─^, *E. coli* collision resulted in a response of tens of pA, which exceeds the instrumental lower limit (a few pA) by one order of magnitude. This concentration is low enough to prevent osmotic cell death but high enough to result in observable signal intensity. The stability of bacterial cells under this condition (20 mM K_4_ Fe(CN)_6_) was confirmed by a cell growth experiment (Supplementary Fig. S2). Next, the particle size-to-UME diameter ratio must be optimized to produce an obvious staircase current^3^,^22^. When the UME diameter was 10 μm, spherical beads with diameters of 500 nm were marginally detectable by the blocking method. As a practical guideline, this ratio (UME diameter to analyte dimension) should be less than approximately 20. Thereby, a 10 μm UME was chosen to detect *E. coli*.

### Luria-Bertani medium test for control experiments

A control experiment was performed using an *E. coli*-free Luria-Bertani (LB) medium which was used for *E. coli* cultivation (Supplementary Fig. S3). No meaningful staircase signal was observed over 500 s in the presence of LB medium which contains several small molecules that are smaller than 340 nm (Supplementary Fig. S4). These small particles did not cause any detectable staircase current decrease under our experimental conditions. Therefore, this system can be used to detect *E. coli* without removing LB, which would be highly experimentally useful.

### Visual confirmation of sigle *E. coli* collision on C-UME

**Figure 3:**
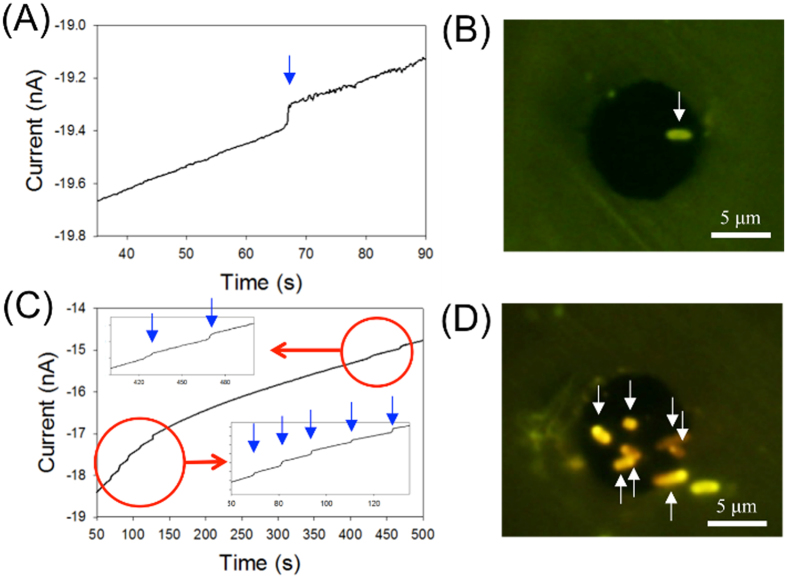
i-t curves and relevant fluorescent images of *E. coli* on C-UME. (**A**) A single staircase current decrease that occurred after single-cell attachment. (**B**) Fluorescent image after single-cell collision experiment. (**C**) Multiple staircase current decreases resulted from multiple *E. coli* collisions. (**D**) Fluorescent image after multiple *E. coli* collisions. The solutions contained 53 fM *E. coli* and 20 mM K_4_Fe(CN)_6_. The C-UME (10 μm diameter) was biased at +0.6 V vs Ag/AgCl. Black circles in the fluorescent images indicate the surface of the C-UME.

To visually confirm the attachment of *E. coli* on the UME surface, enhanced green fluorescent protein (EGFP)-expressing *E. coli* was used. After completing the electrochemical measurements, the electrode was gently washed with distilled water to remove the electrolyte salts. The UME surface was carefully observed using various microscopy techniques to identify the existence of *E. coli* remaining on the UME surface. As shown in Fig. 3A, one collision signal was detected for 90 s. After collecting the electrochemical collision signal, the electrode surface was observed by fluorescent microscopy (Fig. 3B). The green-colored *E. coli* on the UME surface were clearly observabled. Scanning electron microscopy (SEM) also supports the adsorption of *E. coli* at the same position (Supplementary Fig. S5). These microscopic images confirmed that the adsorbed particle on the electrode surface is an *E. coli* and that the cell cannot be easily detached. Therefore, this method can be used to investigate attached single bacteria.

### Detection of multiple *E. coli* collisions on C-UME

The stable adsorption of *E. coli* on the electrode surface eliminated the need to develop an alternative method to stabilize the cells and allowed us to conveniently elucidate the relationship between the number of electrochemical staircase current responses and the actual number of EGFP-expressing *E. coli* that collided with the electrode. An electrode that exhibited multiple collision responses was also investigated. In this case, 7 collision responses were observed over 500 s (Fig. 3C). As shown in Fig. 3D, 7 fluorescent *E. coli* were present on the UME surface (black circles). The number of adsorbed *E. coli* was in good agreement with the number of electrochemical collision responses. Thus, this approach can be used to estimate the number of adsorbed *E. coli* on the UME based on the current transient.

### Simulation for analysing current step decrease and collision frequency

**Figure 4:**
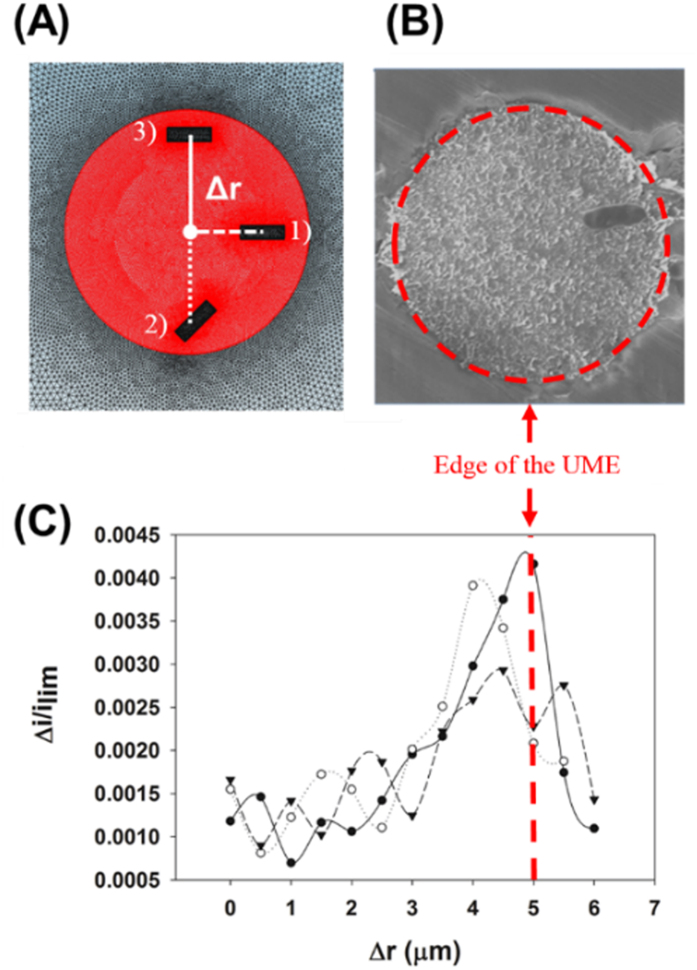
(**A**) In the 3-D simulation domain, *E. coli* was depicted as a cylinder. Red circles indicate the UME surface. Δr is the distance from the center of the UME to the center of the *E. coli.* (**B**) SEM image of *E. coli* attached to the C-UME. (**C**) Simulated relative magnitude of the electrode current (Δi/i_lim_) changes after *E. coli* attachment at various relative distances from the UME center. Solid, dashed, and dotted lines represent three different *E. coli* alignments at the UME, as shown in Fig. 4A.

The magnitude of the staircase current decrease resulting from a single *E. coli* collision was estimated via 3-D Comsol Multiphysics simulations (Fig. 4). Depending on the alignment of the *E. coli*, different current responses would be expected. Thereby, the alignment of the *E. coli* must also be considered (Fig. 4A). The simulation results imply that the relative size of the current step (∆i/i_*lim*_), where i_*lim*_ is the limiting current and ∆r is the distance from the center of the UME to the *E. coli*, tends to increase as the landing position nears the edge of the UME, regardless of the *E. coli* alignment (Fig. 4C). Based on Fig. 4C, the maximum and minimum staircase current heights can be estimated when *E. coli* of a specific size collide with the UME (in our simulation, an *E. coli* was assumed to have a cylindrical shape with a radius of 0.4 μm and a length of 2 μm). The height of the experimentally observed staircase current (83 pA) shown in Fig. 3A is in good agreement with the estimated signal height (75 pA) based on the simulation results shown in Fig. 4C (simulation details are provided in the Supplementary Dataset).

The experimental and theoretical collision frequencies of *E. coli* can be related to the concentration of cells. A 2-D simulation considering the flux of the symmetric UME was performed to obtain the theoretical collision frequency under migrational transport with a steady-state current at the UME (simulation details are shown in the Supplementary Dataset). The model developed here is fairly similar to that described in our previous report^3^,^23^,^24^. Based on this simulation, the expected frequency was ca. 0.47 pM^−1^s^−1^, which was calculated based on the total flux of *E. coli* observed on the UME. The experimental (0.26 pM^−1^ s^−1^) and expected (0.47 pM^−1^ s^−1^) frequencies are on the same order of magnitude. This simulation considered only the diffusional and attractive migrational movement of *E. coli* (between positively developed electric field near UME and negatively charged *E. coli*). This result implies that the complex movement of *E. coli* hardly affects the overall collision frequency.^25^ Opposite electric field (negatively developed electric field near UME) generated by reduction of ruthenium(III) hexamine was tested to confirm the effect of migrational movement of *E. coli* (Supplementary Fig. S6). As shown in Fig. S6, there was no collision signal detected during the measurement time. From these results, we can confirmed that the collision signals under ferrocyanide condition originated from negatively charged *E. coli*. Various *E. coli* concentrations were measured in the same manner (Supplementary Fig. S7).

### Dependency of *E. coli* concentration on collision frequency

The plot in Supplementary Fig. S7 (collision frequency vs. *E. coli* concentration) shows good linearity in the concentration range from 10 to 100 fM. The collision frequency was saturated at 212 fM of *E. coli* because the fully *E. coli*-covered UME, which had reduced electroactive area, exhibited reduced migrational *E. coli* flux to its surface. The concentration of cells in the sample could thus be predicted using the theoretical single-particle collision frequency.

## Conclusions

In this paper, we have demonstrated the electrochemical label-free detection of single living bacteria using the blocking methodology, as was visually confirmed using EGFP-expressing *E. coli*. This blocking method is faster and simpler than the conventional methods used to detect living bacteria in solution. The number of collision responses observed in *i-t* curves correlates with the number of *E. coli* observed via fluorescence microscopy and predicted by theoretical calculations. The theoretical current responses of single *E. coli* collisions were obtained by Comsol Multiphysics simulations. These simulation results are also in good agreement with the experimental results. Therefore, this methodology can be applied to measure the concentrations of specific-sized microbes in solution. By observing the cells adhered to the UME, this method can provide a platform to study the properties of single cell after separation and/or attachment onto the UME surface. This approach can also be applied to study other biomolecules.

## Methods

### Reagents and Materials

Potassium ferrocyanide, ferrocenemethanol (97%), hexane (>95%), toluene (99.9%), isopropyl alcohol (IPA, >99.9%), ethanol (99.9%), and Hexaammineruthenium(III) chloride (98%) were obtained from Sigma-Aldrich. Potassium chloride (99%) was obtained from Fisher Scientific. All chemicals were used as received. Carbon fiber (diameter 10 μm) was obtained from Goodfellow (Devon, PA). Borosilicate capillary tubing (1.5 mm o.d. ×0.75 mm i.d.) was obtained from FHC Inc. (Bowdoin, ME). Millipore water (>18 MΩ cm) was used in all experiments.

### Instrumentation

The electrochemical experiments were performed using CHI900B and 750E potentiostats (CH Instruments, Austin, TX) with a three-electrode cell placed in a Faraday cage. A 1 mm diameter Pt wire was used as a counter electrode, and the reference electrode was Ag/AgCl (3 M KCl). Scanning electron microscopy (SEM) was performed using a Sirion FEI XL FEG/SFEG microscope (FEI Co., The Netherlands) with an accelerating voltage of 2 kV. Fluorescence microscopy images were taken using an Olympus IX71 inverted microscope with a mercury lamp and a CCD camera system (DP70 camera unit and PCI board). Dynamic laser scattering (DLS) data was obtained by Zetasizer Nano ZS (Malvern, Westborough, MA). Nanoparticle tracking analysis (NTA) data was obtained by NanoSight NS300 (Malvern, Westborough, MA). 2D and 3D simulation were performed using Comsol Multiphysics.

### Preparation of Carbon Fiber Ultramicroelectrodes (C-UMEs)

C-UME was prepared following the general procedure developed in our laboratory. Briefly, a 10 μm (diameter) carbon fiber was sealed in a borosilicate glass tubing (1.5 mm o.d. ×0.75 mm i.d.) after rinsing with hexane, toluene, IPA, ethanol, and water. The electrode was then polished with an alumina powder water suspension to a mirror finish. The surface area was checked with standard redox electrochemistry in ferrocenemethanol solution. Before every experiment, all electrodes were polished with alumina (0.05 μm) paste on microcloth pads (Buehler, Lake Bluff, IL) prior to use.

### Expression of EGFP protein

*Escherichia coli* cells (*E. coli*) (DH5α, Enzynomics) were cultivated in Luria-Bertani (LB) medium with kanamycin (50 μg/mL, Merck) at 37 °C with vigorous shaking. EGFP protein was expressed by 0.1 mM IPTG (isopropyl β-D-1-thiogalactopyranoside, Duchefa Biochemie) for overnight. The region of EGFP was amplified from pEGFP-C1 (Clontech Laboratories, Inc.) by polymerase chain reaction (PCR) and cloned into the *NdeI*/*XhoI* sites of protein expression vector, pET-30b(+) (Novagen). The following oligonucleotides were used to perform PCR. (*NdeI*_EGFP_F: GGA ATT CCA TAT GGT GAG CAA GGG CGA GGA GCT, *XhoI*_EGFP_R: CCG CTC GAG C TTG TAC AGC TCG TCC ATG, Bioneer) The concentration of *E. coli* was calculated from Growth curve of EGFP-expressed cells (Supplementary Fig. S8).

## Additional Information

**How to cite this article**: Lee, J. Y. *et al*. Label-Free Detection of Single Living Bacteria via Electrochemical Collision Event. *Sci. Rep.* **6**, 30022; doi: 10.1038/srep30022 (2016).

## Supplementary Material

Supplementary Information

Supplementary Dataset

## Figures and Tables

**Figure 1:**
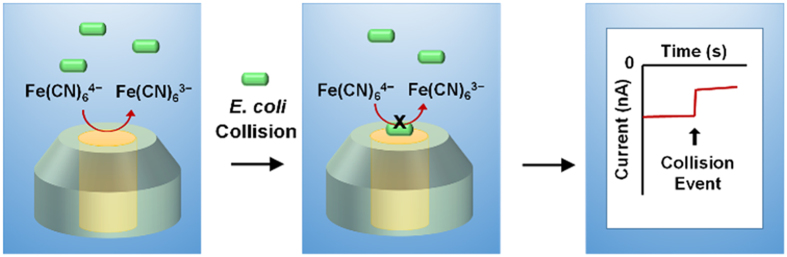
Schematic diagram of *E. coli* detection by collision event on a UME.
