# Supplementary material for: Label-Free Detection of Single Living Bacteria via Electrochemical Collision Event
Source: Sci Rep. 2016 Jul 20;6:30022. doi: 10.1038/srep30022 (PMC4951717; doi:10.1038/srep30022)
Supplement: Supplementary Information [file srep30022-s1.pdf]

Supplementary Information

Label-Free Detection of Single Living Bacteria via Electrochemical Collision Event

Ji Young Lee,[1,†], Byung-Kwon Kim,[2,†,*] Mijeong Kang,[3] Jun Hui Park [4,*]

[1] Korea Advanced Institute of Science and Technology, Department of Chemistry, Daejeon 34141, South Korea

[2] Sookmyung Women's University, Department of Chemistry, Seoul 04310, South Korea

[3] University of Maryland, Fischell Department of Bioengineering, College Park, MD 20742, USA

[4] Chonbuk National University, Department of Chemistry Education and Institute of Fusion Science, Jeonju 54896, South Korea

2D Comsol Multiphysics Simulation

We assume that the UME reaction is one electron oxidation of ferrocyanide ($Fe(CN)_6^{4-} \rightarrow Fe(CN)_6^{3-} + e$). The Nernst-Planck equations, (1), was used to describe the *E.coli* collision event under migration and diffusion:[i]

$$J_i(r,z) = -D_i \nabla C_i(r,z) - u_i C_i(r,z) \nabla V(r,z) \quad (1)$$

where J_i is the flux of each species, D_i is the diffusion coefficient, C_i is the concentration, u_i is the electrical mobility of the ionic species, and V is the electric potential. The space dimension was taken as 2-D axial symmetry in the simulation which applied Nernst-Planck steady-state equation.

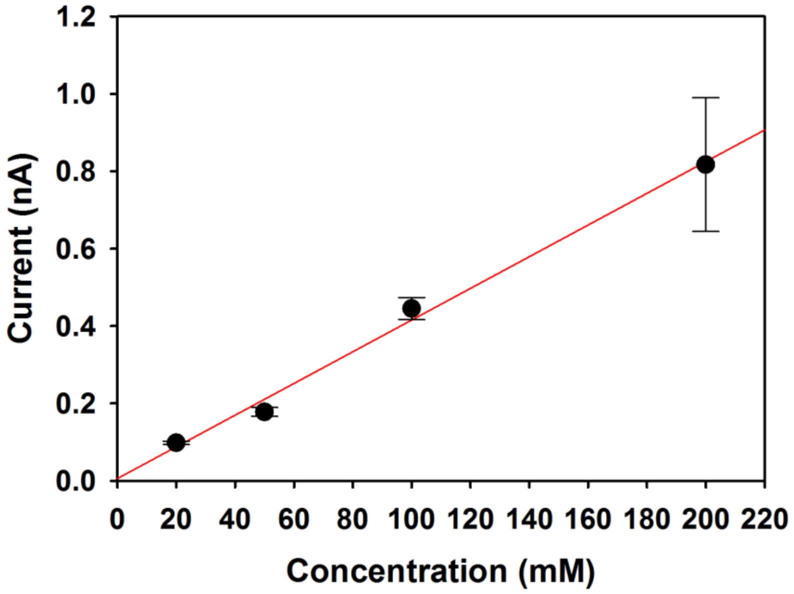

Figure S1. Heights of staircase current responses by single *E. coli* collision on UME as a function of ferrocyanide concentrations. The error bars represent the standard deviations for at least three measurements. The straight line is linear fit of the experimental values.

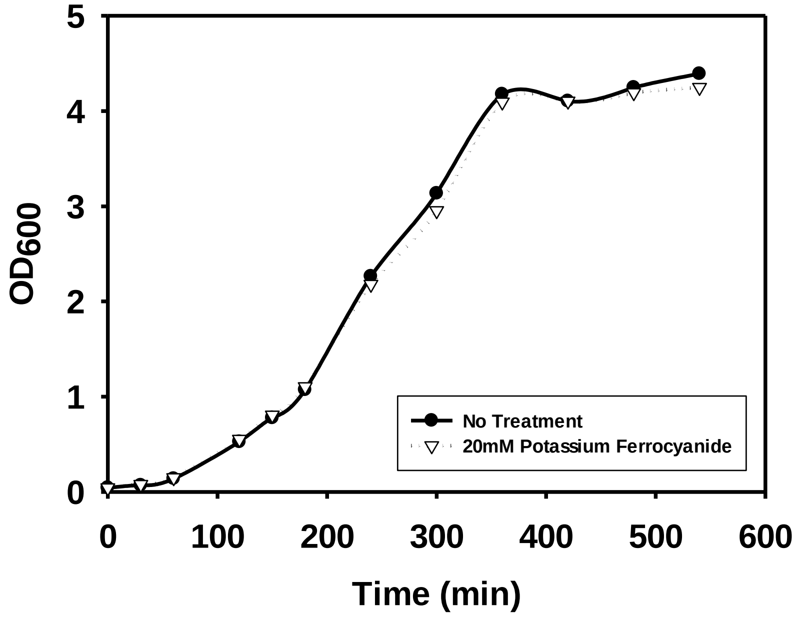

Figure S2. Growth curve of *E. coli* cells after the treatment of 20 mM potassium ferrocyanide (▽) and no (●) treatment for 10 min. Cells at stationary growth phase (OD_{600} = 4.0) were used in this experiment. Overnight culture is diluted to 100-fold. The number concentration of *E. coli* cells in sample is approximately 3.2×10^9 cells/mL (bacterial cell cultures, OD_{600} of 1.0 = 8×10^8 cells/mL).

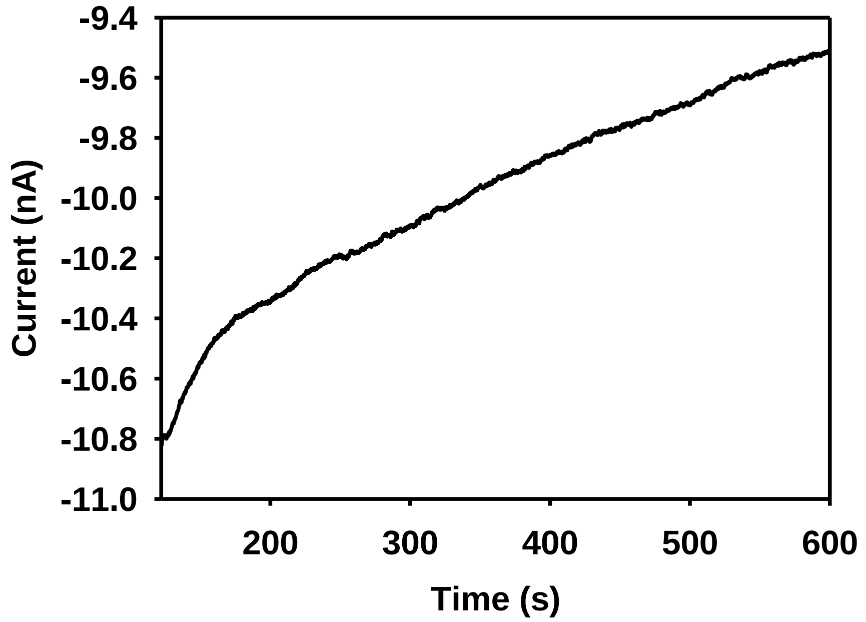

Figure S3. Amperometric i-t curve of the control experiment. The solution contains 20 mM potassium ferrocyanide and LB medium. The C-UME potential was +0.6 V (vs. Ag/AgCl) applied at 0 s.

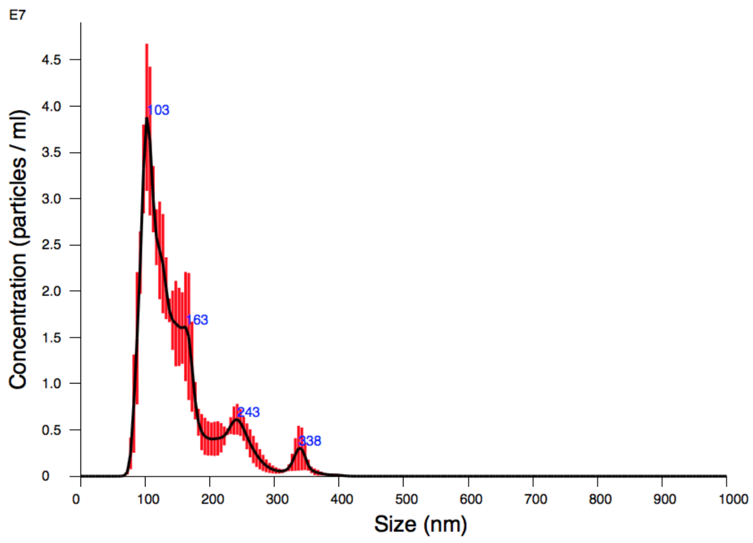

Figure S4. Particle size distribution of small particles in LB medium obtained by NTA

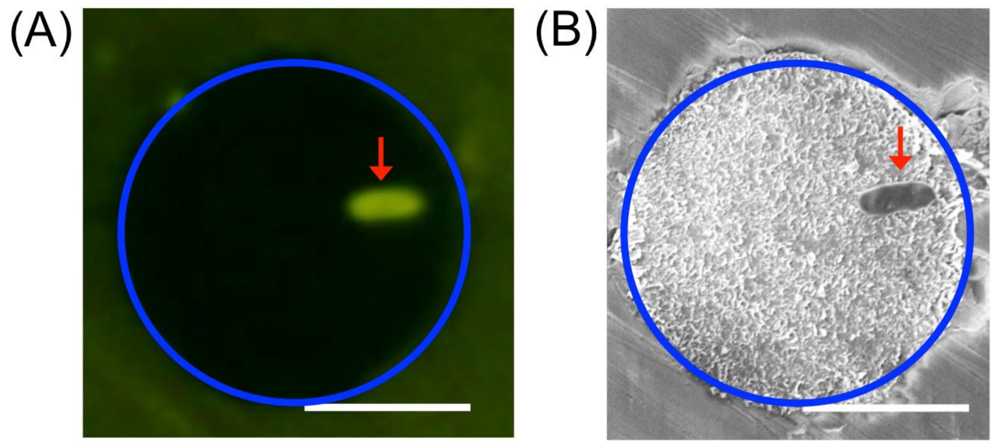

Figure S5. (A) Fluorescence and (B) SEM images of a single *E. coli* on UME surface after collision experiment. The blue circle and red arrow indicate the UME edge and single attached *E. coli*, respectively. The white bar indicates 5 μm.

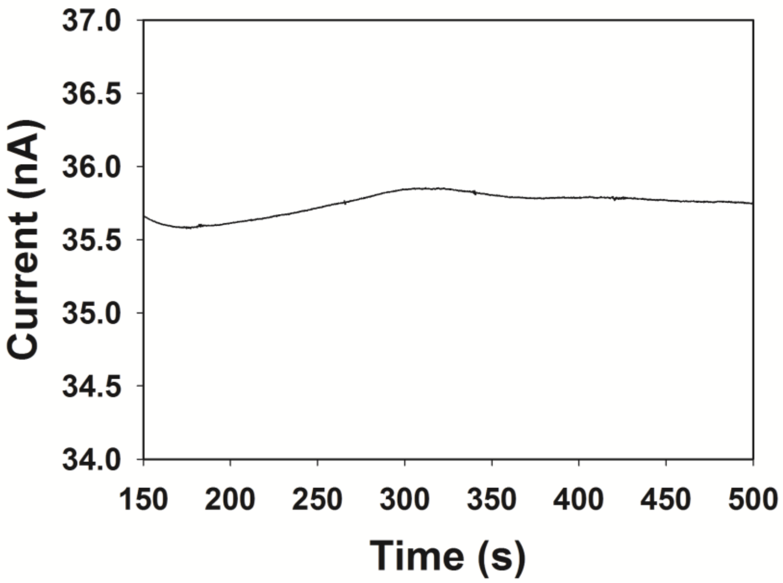

Figure S6. Amperometric i-t curve of the opposite (negative) electric field experiment. The solution contained 20 mM ruthenium(III) hexamine trichloride. 106 fM *E. coli* were added at 100 s. The C-UME potential was -0.5 V (vs. Ag/AgCl) applied at 0 s.

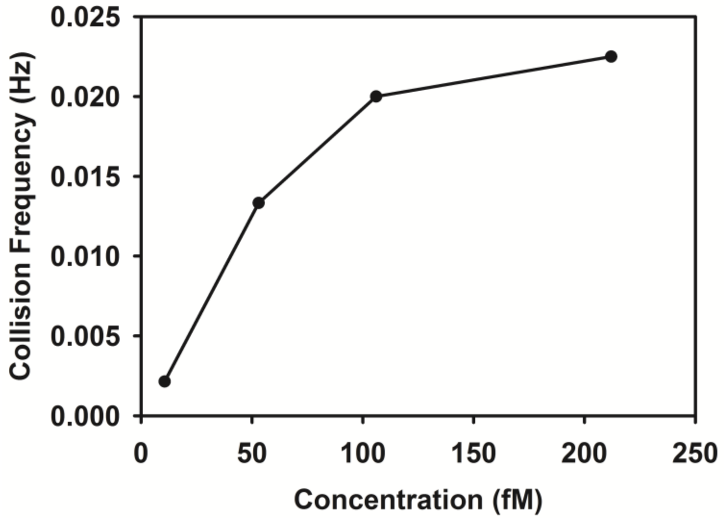

Figure S7. Frequencies of collision event as a function of *E. coli* concentrations.

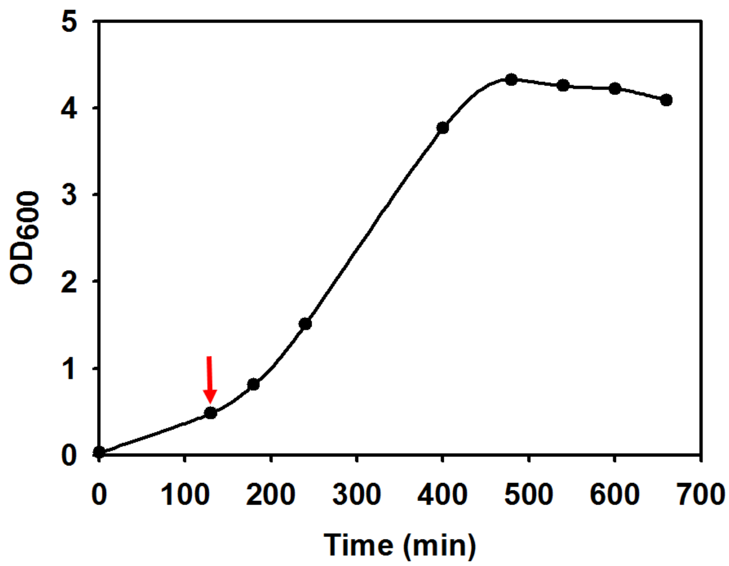

Figure S8. Growth curve of EGFP-expressed *E. coli* cells. Overnight culture is diluted to 200-fold. EGFP expression is induced at the indicated point (red arrow). Cells at stationary growth phase (OD_{600} = 4.0) were used in this experiment. The number concentration of *E. coli* cells in sample is approximately 3.2×10^9 cells/mL (bacterial cell cultures, OD_{600} of 1.0 = 8×10^8 cells/mL).

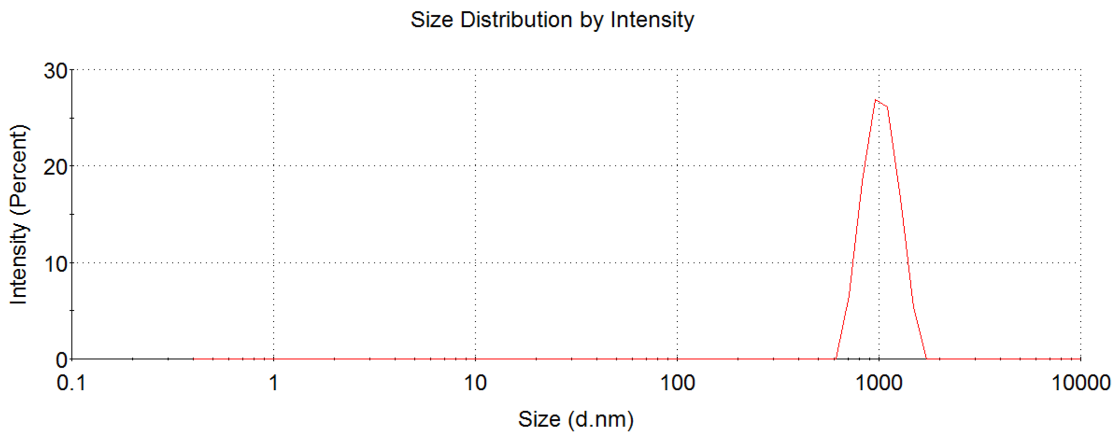

Figure S9. Size distribution of *E. coli* in water obtained by DLS. The average size of *E. coli* is 1037 ± 197 nm.

---

[i] Bard, A. J.; Faulkner, L. R. Electrochemical Methods: Fundamentals and Applications, 2nd ed.; John Wiley and Sons: New York, 2001; Ch. 4, pp 137—140.
